# Pone

**Published:** 1857-05

**Authors:** 


### HALL'S JOURNAL OF HEALTH.

OUR LEGITIMATE SCOPE IS ALMOST BOUNDLESS: FOR WHATEVER BEGETS PLEASURABLE AND HARMLESS FEELINGS, PROMOTES HEALTH; AND WHATEVER INDUCES DISAGREEABLE SENSATIONS, ENGENDERS DISEASE.

VOL. IV.] MAY, 1857. [NO. V.

We aim to show how disease may be avoided, and that it is best, when sickness comes, to take no medicine without consulting an educated physician.

### PONE.

WHO knows what "Pone" is? Is it Greek, Hebrew, or High Dutch? How waters the mouth at its mention, of him who knows its meaning! How longingly and lovingly does the exile from home revert to the happier days when he revelled in "Pones!"—Pones, only to be enjoyed at home, because not produced on a distant soil.

If the reader is not in a hurry, we will begin at the beginning. Some three thousand years ago, there was a nation called Roman, who spoke a language termed Latin. Among many of its beautiful words was one pronounced "*Pono*," answering to our word place or set, to set a thing away, for example; so it was used to mean "*setting*." And as the sun "sets," it came to be employed to designate the place where the sun sets, that is, west, and what occurred there was denominated "western." Milton wrote of "*ponent* winds," that is, "western" winds. As Americans do everything "*for short*," and lop off all redundancies, we have only to drop the last two letters from Milton's word, and thus make "Pone," which signifies "western!"

Having "come up" ourselves among the corn-stalks of Kentucky, almost under the shade of immortal "*Ashland*," made so by its peerless occupant, HENRY CLAY, we ought to know all about "western" things, this "*Pone*" especially. Although of Latin origin, it is more exclusively used by the African race, the "*People of Color*," as the aristocratic among them prefer to be called. They not only employ the term every day of their lives, but they luxuriate in the thing itself, dearer to them than the dram of the drunkard. In short, "Pone," in the mind of the darkey of all ages, sexes, and cliques, symbolizes "*Bread,*" the staff of life. So, when not in a hurry, he asks for a "*Pone of Bread,*" meaning thereby a piece of bread as large as the extended hand, and two or three times as thick, hot as it can be, and made of "corn meal," known in the east as "*Indian meal.*" So a "*Pone*" stands for half a pound of hot bread, made out of Indian corn ground coarse. In other words, a piece of "*western*" *bread,* as it appears in every family, every day of the world. And when you do not see it in any day on the table, you may know you are no longer in Kentucky, or in the west.

The practical point is, "How is a "*Pone of Bread*" made—the real, pure, genuine, original, identical "*Corn Dodger*"?

Not having studied that valuable science, "Cookery," in early life, it was among the lost arts, the manufacture of "Pones," when we came to New York. So we gave up the expectation of ever seeing them on our own table in this latitude. But to come as near it as possible, we keep several of these "Pones" in our office, made years ago on Western soil by *Phœbe,* the cook of our youth. The artistic prints of her great big black fingers are upon them in distinct bas-relief. Besides, we have a pill-box of the "meal" which makes them, thinking that one of these days we will go down to the *Hecker* brothers—their "nether" office, mind, where *flour* is made, the "upper" one being employed as the manufactory of Puseyite theology, "samples" of which are exhibited in the *New-York Churchman* hebdomidally. Whether the theology is as purely white as the flour, we will not decide here—perhaps they are both of a whiteness. Certain it is, that the Hecker flour is the whitest in the world; certain it is, also, that the whiter the flour, the less nutriment—substance—it has. Therefore flour may be so white as to have nothing in it. Then there are things very white which have worse than nothing in them; such as whitened sepulchres, full of raw heads and bloody bones, and things like that. It is possible, therefore, that a theology may be marvellously white and comely to look at, just like treble-refined flour, and have nothing in it valuable, but hiding up all sorts of monstrous heresies and demoralizing dogmas; white, like the flour, to look at, but when fed upon, yields no nutriment, sits heavy on the stomach, causing to the body dyspepsia, and to the mind "*chimeras dire.*" Now this is precisely the reason we never see "*Western*" Indian on our tables. The Eastern is so refined, like the transcendental theology of the fatherland, that it does not answer the purpose for which it was made, that is, of a wholesome, nutritious, healthful food. Thus too is it, that to make it swallowable, to make it at all digestible, every recipe almost that we have ever seen, requires a dose of physic to be put in the corn meal of the *East*, to prevent it from killing those who eat it habitually. Only think of the absurdity of the thing—sending to a druggist every time corn bread is wanted on our table.

Look at our miserable *cookery books!* Why, it is impossible to spread a table with ordinary provisions without having some article floating in grease, fired with spices, or saturated with salts or tartar, or other nauseating drug.

We do not go all lengths with the water cure advocates and vegetarian fanaticisms, but we do say most uncompromisingly, that only their cook-books ought to be tolerated. Half an age would be added to human life in half a century, if our food was prepared by their directions, and an animal diet in moderation, were added thereto.

At this present writing, one of the tastiest of Kentucky's daughters, and one of the tidiest of housekeepers, is with us for a "*spell*," and talking with her about *lang syne*, the leeks and onions of Egypt, we bethought us of our old time "*Pone*," how natural it would look, how home-like, with our colored servants, to see an old-fashioned *Corn Dodger* on our table. Now a real Kentucky daughter, with the blessing of an old-fashioned Kentucky mother, can put her hand to anything "in reason," from the sculling of a yawl across the Mississippi, to the trimming of a bonnet; from the fitting of a dress to the driving of a "pair;" from the waltz of a ball-room—no, not exactly that; our conscience will not allow us to sacrifice truth to any antipodism or a parallel. The ball-room is not patronized by the best Kentucky families. Well, we will let that go. A genuine Kentucky lass can make a dress or wash the dishes, and cause you to feel all the time that you must keep your distance You take a liberty at your peril, whether at the wash-tub or the piano. The cleanliest, tidiest kitchen we ever saw, or ever expect to see, was that kept by the daughter of a Kentucky mother. Surely "*there's nothing like leather.*" Look at it! see what drafts the great East makes on her distinguished western sister for recruits to supply the places of her own fallen magnates. An incident of May, 1856, may show our meaning, how Kentucky greatness is drifting to the metropolis of the western hemisphere. The acknowledged leader of *Young Presbytery*, the Rev. Thornton A. Mills, was a Kentuckian, since called to occupy in New York one of the most important and responsible posts of that church. The "*lion*" of the Old School party at the same time was a Kentuckian, and the "*lamb*" of the Assembly, the amiable and gifted *Humphrey*, among the next in might and mind of that congregated greatness, was a Kentuckian, by reason of long residence there, while beyond all others, and deservedly so, in professional skill in his line of *medical surgery*, is another who hails from Kentucky—Dr. Bodenhamer; and as to ourself, see how the coach-wheels fly: our Journal is pronounced "one of the most useful family periodicals we have ever seen," and its editor, "*one of the most practical men of the age. He tells us how to do almost everything that is worthy of being done at all.*" On the Atlantic shore, a great and good divine writes, "You are a blessing," and scarcely had his kindly words died away, when there comes from beyond the *Sierra Nevada*, just from the shores of the great Pacific sea, from a name not below the highest in goodness and scholarship and influence, "*In behalf of mankind I thank you for your Journal*," while multitudes of minor minds strike the same string! Contemplate how we apples navigate!!

But, after all, what are we? If this hour we were stricken down in death, the hum of this city would not be a note the less loud; our majestic Hudson would flow as placidly on to the boundless sea, each bright particular star would twinkle as lovingly out of the great blue deep above, as silvery would the shining moon dance upon the ocean's wave, and brightly as ever would the sun beam upon the busy world, beyond our own solitary home; and even there how small, how short the change—not one smile the less would dimple the cheek of our three year old; our little Alice, to whom "fader" is to-day the all in all, would turn to "mudder" to-morrow, all oblivious that that father had ever lived; and wifey, too, with a few sad remembrances, would, in an o'er brief space, begin to woo and win as once before she wooed and won, spite of the ready vow of to-day, she "*never would—no,* NEVER!"

Really, man and all his struttings and mimicry, reminds us of a frequent sight of our childhood's summers—of two big black bugs laboring in the public road with might and main to roll together a ball of earth. No Napoleon ever struggled harder to crush a crown; no Cæsar was animated with a more absorbing desire to wear the diadem of empires, when presently a tremendous wagon-wheel rolls along and crushes bug and ball of earth to countless atoms, while time and tide, and sun and star move as they had moved before, nothing changed but the bug and his cherished ball of dust!

Said we to the handy Kentuckian aforesaid, Suppose we have a home breakfast in the morning; the pure cream and the fresh butter we will engage to supply, if you will promise to have the "*Pone*" forthcoming. And it was done. We inquired how. All was luminously explained, but sight was always more valuable to us than sound, an easier teacher by far; so we *encored* the operation, and thuswise was it performed:

A quart of Indian meal was put in a wooden bowl with as much salt as would be taken up with the thumb and fingers, that is, about a teaspoonful; then add as much sweet milk as will make it up into adherent dough, of which take up a double handful, laying it over on one hand and thus carry it to the pan or skillet for baking, turn it in with one pat of the hand, and so on, until the vessel is full, and with a good heat, let it remain until the crust is a yellowish brown; put it on the table piping hot, press it open, lay in a large lump of grass butter just made (if you can get such a thing), and it is ready for demolition.

Corn bread is best if eaten while it is hot; it becomes sodden as it cools. The milk supersedes the use of lard or butter; no water is needed, although many use butter and water instead of milk; but the true constituents of a *Pone* of bread are meal, milk, salt, nothing else. If you add eggs, it becomes Johnny cake, and is no longer a "*Pone of bread.*"

A more simple, healthful, nutritious, and agreeable article of bread, is, in our opinion, never made than the one we have described. The roughness of the meal particles gives the advantages of brown bread; its natural sweetness makes sugar or molasses unnecessary; while the sweet milk answers all the purpose of soda or cream of tartar.

It is important to put it into the pan for baking, the instant it is made, and to have it baked as rapidly as practicable without burning the crust.

The Indian meal of the East is ground so fine that the bread will be more or less sodden without soda. If it were a coarser article there is no reason why we should not have as good corn bread in New York as in the garden of the West; the bread would be "light" without medical aid.

And all these five pages about a "Pone!"

